# The association between voluntary work and health care use among older adults in Germany

**DOI:** 10.1186/s12913-019-3867-x

**Published:** 2019-01-15

**Authors:** Maike Flennert, Hans-Helmut König, André Hajek

**Affiliations:** Department of Health Economics and Health Services Research, Hamburg Center for Health Economics, University Medical Center Hamburg-Eppendorf, Martinistr. 52, 20246 Hamburg, Germany

**Keywords:** Volunteering, Volunteer, Health care use, Health care utilization, GP visits, Specialist visits, Hospitalization, Longitudinal study

## Abstract

**Objective:**

While most studies focused on the relation between volunteering and health-related outcomes, little attention has been given on the association between volunteering and the use of health care services. Thus, with this analysis we aimed at exploring whether and how the voluntary work of older adults is related to the utilization of health care services in Germany.

**Methods:**

The analysis was based on data from the German Ageing Survey (DEAS), a nationally representative, longitudinal study of the German population aged 40 years and older. Focusing on volunteering, data from the waves 2002, 2008 and 2011 was used. Voluntary work in groups and organizations (yes/no) was used as explanatory variable. To quantify health care utilization, visits to general practitioners and specialists as well as nights in the hospital in the past 12 months were used. Fixed effects regressions were applied to estimate the association between volunteering and the outcome variables.

**Results:**

Regressions revealed that the onset of volunteer involvement was associated with an increase in specialist visits, whereas volunteering did not affect visits to general practitioners and the probability of hospitalization significantly.

**Conclusion:**

Our findings emphasize the relation between volunteering and specialist visits. Future research is needed to examine the impact of volunteering on health care use, taking more detailed information regarding the specific context of volunteering as well as personality factors and personal background into consideration. This might be reasonable in advancing the knowledge about this association and in developing planned interventions.

**Electronic supplementary material:**

The online version of this article (10.1186/s12913-019-3867-x) contains supplementary material, which is available to authorized users.

## Introduction

*Volunteering* among older adults has become increasingly important in order to overcome challenges resulting from an aging population [1]. In light of this, worldwide policy interest has risen in recent years and different organizations and governments advocated the acceptance of volunteering to strengthen civic engagement [2, 3].

In the present study, we focused on formal volunteering and excluded informal volunteering which involves activities that are provided to relatives, neighbors or friends [4]. Formal volunteering is typically organized by public and non-profit organizations as well as religious associations and governmental programs. It usually entails a broad variety of activities, such as tutoring, mentoring or giving technical advices and support [1, 5]. The growing attention towards volunteering for older individuals stems from its potential to provide not only a meaningful contribution to society but also to the volunteers themselves. On the one hand, health status of older individuals has improved in recent decades and future elderlies will be more active and independent than today [6]. Thereby, these healthy and more active older adults constitute an increasing reservoir of human and social capital that can be used to contribute a valuable service to society, for example, through volunteering [7, 8]. On the other hand, volunteer involvement accounts for a way to improve the health and well-being of the volunteers [9]. This relationship between volunteering and health as well as well-being of older adults has been identified in several cross-sectional studies [10–12]. Furthermore, a number of longitudinal studies have shown that the voluntary work of older adults positively affects health [13–21].

While the studies cited above demonstrate the impact of volunteering by mainly focusing on health-related outcomes, studies investigating potential effects on health care utilization are sparse. To our knowledge, only one recent study by Kim and Konrath [22] investigated the association between volunteering and patterns of health care utilization in America. Regarding the examined outcome measures, the scientists found that volunteers had a greater probability of using preventive health care services as well as a decline in the number of nights spent in the hospital compared to non-volunteers. There was no association between volunteering and the frequency of doctor visits [22]. Despite these initial findings towards an association between volunteering and health care utilization, potential effects of volunteering on health care utilization remain an open question and need more investigation. Additionally, an analysis of the relationship between volunteering and the use of health care services in Germany does not yet exist. This might be reasonable due to existing differences between the American and the German health care systems.

In Germany, health insurance is covered by statutory health insurance (SHI) as well as private health insurance (PHI). Approximately 90% of the population is insured via the SHI, which is characterized as a payroll tax financed system according to the principle of solidarity. Family members are insured without additional payments. The other 10% of the German population is covered by PHI. People, such as civil servants, self-employed, and employees above a certain income threshold can choose between SHI and PHI. In contrast to SHI, private insured must pay risk-adjusted payments according to their age and state of health. All insurees from both types of health insurance are entitled to comprehensive health care and can consult outpatient specialists without the need of referral from general practitioners (GPs). From 2004 to 2012, statutory insured had to pay a small copayment rate for using services of outpatient physicians. Patient hospital admissions require referral by outpatient physicians but in the case of an emergency, hospitals are obligated to provide care to those in need. Further information about the German health care system can be found elsewhere [23].

The aim of the study was to explore whether and how the voluntary work of older adults is related to the utilization of health care services in Germany. Compared to other countries, in Germany the fraction of people consulting a GP or a specialist is very high [24]. This causes an enormous financial burden to the German health care system. Thus, it is important to identify the determinants of health care utilization.

Consequently, with this study we contribute to the existing research by receiving a deeper understanding of the relationship between volunteering and health care utilization of individuals. This is necessary for this area to advance and to address the mentioned challenges resulting from an aging population by organized interventions. Based on previous studies that found an association between volunteering and better health and well-being of older adults, we expect that individuals starting volunteering are in turn less likely to use health care services. Consequently, we hypothesize that the onset of volunteering among older adults in Germany leads to less GP visits and less specialist visits as well as to a reduction in hospitalization.

## Methods

### Sample

Data for this analysis were derived from the German Ageing Survey (DEAS – “Deutsches Alterssurvey”) which was conducted as a nationwide representative cross-sectional and longitudinal study of the community-dwelling population aged older than 40 years in Germany. The survey is funded by the German Federal Ministry for Family Affairs, Senior Citizens, Women, and Youth (BMFSFJ). Since 2002, the study is organized by the German Centre of Gerontology (DZA – “Deutsches Zentrum für Altersfragen”). The cross-sectional baseline samples have been set up on a recurring four-year basis starting in 1996 and ending in 2014. These baseline samples were stratified by gender, age as well as region (Eastern/Western Germany) and randomly selected from population registers within municipalities. If a written permission was given, participants from the baseline samples were re-contacted to take part in additional waves. Data collection for panel assessments were performed in 2002, 2008, 2011, 2014. The sixth wave (2017) is currently conducted.

The objective of the DEAS is to provide a unique database that enables researchers to study the multifaceted living conditions of the German population aged older than 40 years as well as the consequences of aging on individuals and processes of social transition. Therefore, participants were asked questions regarding their socio-demographic circumstances, their living conditions as well as different topics related to aging by using oral face-to-face interviews. These personal interviews took place at the participants’ homes and were carried out by trained interviewers organized as Computer-Assisted Personal Interview (CAPI). At the end of the interview, respondents were asked to fill out a standardized questionnaire that covered, for example, subjective attitudes as well as psychological topics and health.

Due to data availability in the outcome measures and our variable of interest, we restricted the analyses to the second (2002), third (2008) and fourth (2011) waves. Differences between the sample sizes were due to different recruitment approaches. In 2002, 2008 and 2011, individuals who had participated in previous waves were asked for a re-interview. New study participants were solely included in 2002 and 2008. The overall sample size in 2002 was 5194 individuals (of whom 3670 were new and 1524 were re-interviewed) and in 2008 8200 individuals (of whom 6205 were new and 1995 were re-interviewed). In 2011, 4854 individuals took part in the survey interviews (of whom 0 were new and 4854 were re-interviewed). Response rates were 38% in 2002, 38% in 2008 and 56% in 2011. In total, the response rates of the DEAS were consistent with other German surveys, but rather low compared to other European studies on aging [25]. Time or health restrictions as well as refused re-participation were the most common reasons for missing follow-up data [26]. More details of the DEAS have been reported elsewhere [27]. The study was conducted in accordance with the ethical standards of the Helsinki Declaration. Small incentives were provided to all individuals who participated.

### Variables

#### Dependent variables

The use of health care services was measured retrospectively by means of inpatient and outpatient services for 12 months preceding the interview. Outpatient services were assessed by the *number of GP visits* as well as the *number of specialist visits* (house calls included). The variable specialists comprise several medical specialties, which are reported in Additional file 1. The corresponding number of GP and specialist visits was measured as “never”, “once”, “2–3 times”, “4–6 times”, “7–12 times”, or “more often” (open answer). Following Bock et al. [28], it was recoded as “never” = 0; “once” = 1; “2–3 times” = 2.5; “4–6 times” = 5; “7–12 times” = 9.5; and “more often” = 13. Regarding the inpatient sector, the *number of days in hospital* was assessed. The question on hospitalization was treated as a binary variable (1 = “at least one night in hospital”; 0 = “not one night in hospital”).

Mental health services were not investigated separately in our study, as their percentage of usage is quite small (Neurologist/Psychiatrist services in 2002: 6.49%, in 2008: 5.84%, in 2011: 10.42%). Furthermore, visits to psychotherapists and radiologists were not considered due to reasons of data availability. In our study, we did not differentiate whether the type of ward was psychiatric or somatic in the hospital.

#### Independent variables

Our key explanatory variable was *voluntary work in groups and organizations*. The respondents were asked if they execute an honorary office in the groups or organizations in which the person is a member. Response choice of the volunteer variable was binary (1 = “yes”; 0 = “no”).

To investigate the study hypothesis, various variables were chosen to be included in the regression models as an alternative way to potentially explain the relationship between volunteering and health care use among older adults. Variables were selected based on the theoretical framework by Andersen’s behavioral model, preceding research results as well as theoretical interest [29, 30].

The Andersen’s behavioral model is one of the most widely acknowledged models to identify determinants that could plausibly be associated with health care use [30]. It categorizes three basic individual components of health care use: *Predisposing factors* (socio-demographic and health-related belief characteristics, such as age, gender, education and health attitude), *enabling resources* (such as income and social insurance status) as well as *need* for health care (perceived and evaluated state of health) [29].

Regarding *predisposing factors* age, gender (male/female), marital status (married, living together with spouse, others [married, living separated from spouse; divorced; widowed; never married]), employment status (working; retired; other: not employed) and educational level were included. Educational level was categorized according to the International Standard Classification of Education (ISCED-97) [31] scale with three categories: low (ISCED 0–2), medium (ISCED 3–4) and high (ISCED 5–6).

*Enabling resources* covered the (log) monthly equivalent net income in Euro (according to the new OECD equivalence scale) and self-rated accessibility of doctors and pharmacies (1 = “there are enough doctors and pharmacies in the vicinity”; 0 = “there are not enough doctors and pharmacies in the vicinity”).

Regarding *need factors*, self-rated health and morbidity were measured. Self-rated health was quantified by using five states ranging from 1 = “very good” to 5 = “very bad”. To assess morbidity, the number of chronic diseases was recorded which was adapted from the Charlson Comorbidity Index [32]. Furthermore, lifestyle factors, such as current smoking status (1 = “currently smoking”; 0 = “currently not smoking”) and self-reported body mass index (BMI) were included. BMI thresholds were classified according the World Health Organization (WHO): underweight (BMI < 18.5 kg/m^2^), normal weight (18.5 kg/m^2^ ≤ BMI < 25 kg/m^2^), overweight (25 kg/m^2^ ≤ BMI < 30 kg/m^2^), and obese (BMI ≥ 30 kg/m^2^) [33].

### Statistical analyses

To estimate the impact of volunteering in groups and organizations on the use of health care services, FE regressions were used. As a special feature, FE regression models allow for the association between time-constant factors and the explanatory variables. Under the assumption of strict exogeneity, FE regressions lead to consistent estimates [34]. In contrast, techniques such as the pooled ordinary least square (POLS) or the random effects (RE) would lead to inconsistent estimates when unobserved factors and the explanatory variables are correlated. Time-constant unobserved factors, such as gender or genetic disposition, are a widespread topic, especially in social sciences that need to be considered within modern research [35].

Our choice towards the FE specification has been confirmed by performing the Hausman test [36]. It basically investigates whether there is an association between the unobserved time-constant factors and the explanatory variables. The null hypotheses (they are not associated) were rejected for all outcome measures. Therefore, FE regressions were favored against RE regression models and used within this analysis (see Additional file 2).

By removing all time-constant factors, FE estimates are based solely on changes *within* individuals over time (intra-individual changes). As a consequence, the FE estimator is not biased by time-constant unobserved heterogeneity. It is also called “within-estimator” and enables to estimate causal effects by comparing intra-individual changes (with certain restrictions). Since all between-unit variation is eliminated, time-constant variables cannot be estimated in FE regression analysis. Nevertheless, these variables can be used for descriptive purposes and in terms of moderator variables in sensitivity analysis [35].

To estimate the predictors of *GP and specialist visits*, we conducted a FE Poisson regression, which is a commonly used model for measuring count-data [34]. As suggested by Stock and Watson [37], cluster-robust standard errors (SE) were used in order to avoid substantial underestimation of the true standard errors. To estimate the predictors of the binary outcome variable *hospitalization*, a conditional FE logistic regression was applied [38].

For sensitivity analysis, the main model was extended by including an interaction term composed of level of educational status and volunteering (volunteering x education). The underlying idea was that the impact of volunteering on the use of health care services might differ by educational level [39]. As there is evidence that in Germany formal volunteering is more likely performed by men [40], we further tested whether gender-specific links between volunteering and the outcome variables exist. Therefore, we included a respective interaction term (volunteering x gender).

The proportion of missing values was smaller than 3% for all explanatory variables, except for the variable income which had less than 6% missing values. To achieve statistical significance, explanatory variables need to reach a *p*-value smaller than 0.05. Statistical analyses were conducted using Stata 14 [41].

## Results

### Sample characteristics

The pooled (2002, 2008 and 2011) median of GP visits and of specialist visits was 2.5 and 5.5 consultations in the 12 months preceding the interview. In this time span, 88.9% of the individuals consulted a GP and 95.3% a specialist at least once. Moreover, about 18.6% spent one or more nights in the hospital during the year preceding the interview.

Table 1 shows detailed pooled descriptive characteristics of the individuals for all three outcome variables. As already mentioned, we were interested in intra-individual changes over time and only the individuals of the sample who had changes in the outcome variables between the waves 2002, 2008 and 2011 were included.

**Table 1:** Sample characteristics of the individuals included in the FE regressions (waves 2–4, pooled)

		Hospitalization (*N* = 2186)	GP visits (*N* = 6586)	Specialist visits (*N* = 7068)
Time constant variables (not included as independent variables in FE regressions)	Female: *N* (%)	999 (45.7)	3188 (48.4)	3408 (48.2)
	Low education (ISCED-97; 0–2): N (%)	175 (8.3)	462 (7.3)	481 (7.1)
	Medium education (ISCED-97; 3–4): *N* (%)	1130 (53.5)	3293 (51.9)	3463 (50.8)
	High education (ISCED-97; 5–6): *N* (%)	809 (38.3)	2595 (40.9)	2872 (42.1)
Predisposing factors	Married, living together with spouse: *N* (%)	1624 (74.3)	4931 (74.9)	5290 (74.8)
	Working: *N* (%)	612 (28.0)	2419 (36.7)	2615 (37.0)
	Retired: *N* (%)	1345 (61.5)	3406 (51.7)	3638 (51.5)
	Other: not employed: *N* (%)	229 (10.5)	761 (11.6)	815 (11.5)
	Age (in years): mean (SD)	65.1 (10.8)	63 (11.0)	63 (11.0)
Enabling resources	Monthly equivalent net income in Euro: mean (SD)	1734.6 (1436.6)	1728.7 (1436.1)	1786.2 (1636.9)
	Enough doctors and pharmacies: *N* (%)	1849 (84.6)	5456 (82.8)	5891 (83.4)
Need factors	Underweight: *N* (%)	20 (0.9)	42 (0.6)	45 (0.6)
	Normal weight: *N* (%)	742 (33.9)	2431 (36.9)	2665 (37.7)
	Overweight: *N* (%)	982 (44.9)	2828 (42.9)	3021 (42.7)
	Obesity: N (%)	442 (20.2)	1285 (19.5)	1337 (18.9)
	Self-rated health (from 1 = “very good” to 5 = “very bad”): mean (SD)	2.6 (0.8)	2.4 (0.8)	2.4 (0.8)
	Number of chronic diseases: mean (SD)	2.9 (1.9)	2.4 (1.8)	2.4 (1.8)
	Currently smoking: *N* (%)	338 (15.5)	1086 (16.5)	1157 (16.4)
Volunteering	Voluntary work in groups and organisations: *N* (%)	419 (19.2)	1390 (21.1)	1503 (21.3)

Notes: The numbers in the variable educational status do not sum up to 2186 / 6586 / 7068 due to missing values

*GP* = General practitioner, *ISCED* = International Standard Classification of Education, *SD* = Standard deviation

FE regression with GP visits as outcome measure was based on a total number of *n* = 6586 participants. Regarding the time-constant variables, which are not included in FE regressions, 48.4% of the participants were female and most participants had a medium level of education (51.9%). Regarding these participants which are included in FE regression analyses, mean age was 63 years (± 11 years), with a range of 40 to 95 years. More than half of the sample consisted of retired individuals (51.7%), was married, living together with a partner/spouse (74.9%) and reported adequate accessibility of doctors and pharmacies (82.8%). Furthermore, mean monthly equivalent net income was €1728.7 (± €1436.1). Most of the individuals had overweight (42.9%) or normal weight (36.9%). The mean self-rated health was 2.4 (± 0.8) and the mean number of chronic diseases was 2.4 (± 1.8). Only 16.5% were currently smoking. As for volunteering, 21.1% of the individuals reported to be involved in volunteering.

In total, descriptive characteristics for participants included in FE regression analysis with specialist visits as outcome measure were similar. For individuals with hospital stay as outcome variable descriptive statistics differed slightly. For example, mean age was 65.1 years (± 10.8 years) and the mean number of chronic illnesses was 2.9 (± 1.9). Concerning the voluntary work, 19.2% of participants volunteered (21.3% of individuals with specialist visits as outcome measure). However, the total number of observations mainly differed among the three outcome variables due to the different number of observations with intra-individual changes over time in the outcome variables. Thus, FE regression analysis with specialist visits as outcome measure was based on a total of *n* = 7068 individuals, and was based on a total of *n* = 2186 individuals with hospital stays as outcome measure.

To avoid imprecise regression estimates resulting from time-varying variables with only little within variation [42], the variables were tested before including them in the FE regressions. However, our data showed sufficient within-unit variation in order to include all variables in the FE regression models.

### Correlations

To get a more comprehensive picture of our data, pairwise cross-sectional correlations were computed. To account for the problem of multiple comparisons, Bonferroni-adjusted significance levels were used. Detailed results are reported in Additional file 3. Regarding volunteering and the three outcome measures, we found that volunteering was negatively correlated with GP visits (*r* = − 0.06, *p* < 0.001), whereas it was not significantly associated with the probability of hospitalization and specialist visits. Among the other explanatory variables, the highest correlation was found between self-rated health and chronic diseases (*r* = 0.44, *p* < 0.001). There was no correlation value higher than +/− 0.50 which indicates that the correlation level of the variables within our analyses can be assessed as low [43].

### Regression analysis

Table 2 depicts the results of the FE poisson regressions with GP and specialist visits as outcome variables. Adjusting for control variables, regression results for *GP visits* indicated that the number of GP visits was not significantly affected by changes from “no volunteer involvement” to “volunteer involvement”. Among the predisposing factors, the number of GP visits significantly increased with changes in employment status from “working” to “retired” (β = 0.15, *p* < 0.01) and from “working” to “not employed” (β = 0.18, *p* < 0.001). Regarding enabling resources, none of the variables caused a significant change in the number of GP visits. Lastly, results of the FE regression showed that among need factors, GP visits significantly increased with worse self-rated health (β = 0.17, *p* < 0.001) as well as with an increase in the number of chronic diseases (β = 0.06, *p* < 0.001).

**Table 2:** Results of FE poisson regressions (with GP and specialist visits as outcome measures; waves 2–4)

	Independent variables	GP visits	Specialist visits
Predisposing factors	Other marital statuses (ref.: Married, living together with spouse)	0.0178 (0.0563)	−0.0050 (0.0578)
	Retired (ref.: Working)	0.1458** (0.0507)	−0.0118 (0.0455)
	Other: not employed	0.1822*** (0.0521)	0.0847+ (0.0449)
	Age (in years)	−0.0054+ (0.0032)	−0.0086*** (0.0029)
Enabling resources	(Log) monthly equivalent net income	0.0546 (0.0409)	0.0473 (0.0377)
	Self-rated accessibility of doctors and pharmacies (ref.: No accessibility)	0.0172 (0.0306)	0.0052 (0.0291)
Need factors	Underweight (ref.: Normal weight)	0.2651+ (0.1504)	−0.0557 (0.1597)
	Overweight	−0.0123 (0.0421)	−0.0649+ (0.0384)
	Obesity	−0.0416 (0.0612)	−0.0443 (0.0621)
	Self-rated health (from “very good” to “very bad”)	0.1706*** (0.0180)	0.1449*** (0.0175)
	Number of chronic diseases	0.0619*** (0.0094)	0.0596*** (0.0091)
	Currently smoking (ref.: Currently not smoking)	−0.0684 (0.0652)	−0.0782 (0.0570)
Volunteering	Volunteer involvement (ref.: No volunteer involvement)	0.0066 (0.0360)	0.0625* (0.0300)
	Observations	6586	7068
	Number of individuals	3013	3233

Notes: Beta coefficients were reported; Cluster-robust standard errors in parentheses

*Ref.* = Reference, *GP* = General practitioner

*** *p* < 0.001, ** *p* < 0.01, * *p* < 0.05, + *p* < 0.10

For *specialist visits*, results of the FE poisson regression indicated that specialist visits increased significantly with changes from “no volunteer involvement” to “volunteer involvement” (β = 0.06, *p* < 0.05), after adjusting for control variables. Among the predisposing factors, it was shown that the number of specialist visits decreased with higher age (β = − 0.01, *p* < 0.001). Concerning enabling factors, none of the variables affected specialist visits on the statistical significance level. Among the need factors, the number of specialist visits increased with decreased self-rated health (β = 0.14, *p* < 0.001). Moreover, specialist visits increased with the number of chronic diseases (β = 0.06, *p* < 0.001).

Regarding *hospitalization*, adjusting for potential confounders, the conditional FE logistic regression showed that changes from “no volunteer involvement” to “volunteer involvement” did not affect the probability of hospitalization significantly. Regarding predisposing factors, the probability of being hospitalized increased with changes from employment status “working” to “not employed” (OR:1.79, *p* < 0.05). Moreover, the probability of hospitalization increased with increasing age significantly (OR: 1.04, *p* < 0.01). Among enabling resources, results revealed that none of the variables reached the chosen statistical significance level of 0.05. With respect to need factors, the probability of spending a night in the hospital rose with worse self-rated health (OR: 1.74, *p* < 0.001). Lastly, the probability of being hospitalized increased with changes in the smoking status from “currently not smoking” to “currently smoking” (OR: 0.45, *p* < 0.01). Detailed results are shown in Table 3.

**Table 3:** Results of conditional FE logistic regression (with hospitalization as outcome measure; waves 2–4)

	Independent variables	Hospitalization (Ref.: No)
Predisposing factors	Other marital statuses (ref.: Married, living together with spouse)	0.679 (0.374–1.232)
	Retired (ref.: Working)	1.075 (0.687–1.683)
	Other: not employed	1.788* (1.132–2.826)
	Age (in years)	1.038** (1.009–1.067)
Enabling resources	(Log) monthly equivalent net income	0.796 (0.552–1.149)
	Self-rated accessibility of doctors and pharmacies (ref.: No accessibility)	1.188 (0.888–1.590)
Need factors	Underweight (ref.: Normal weight)	1.466 (0.377–5.695)
	Overweight	0.936 (0.646–1.356)
	Obesity	0.930 (0.539–1.605)
	Self-rated health (from “very good” to “very bad”)	1.741*** (1.493–2.031)
	Number of chronic diseases	1.047 (0.968–1.133)
	Currently smoking (ref.: Currently not smoking)	0.445** (0.262–0.757)
Volunteering	Volunteer involvement (ref.: No volunteer involvement)	1.181 (0.841–1.657)
	Observations	2186
	Number of individuals	964
	Pseudo R^2^	0.07

Notes: Odds ratios were reported; 95% CI in parentheses

*Ref.* = Reference

*** *p* < 0.001, ** *p* < 0.01, * *p* < 0.05, + *p* < 0.10

For sensitivity analysis, a volunteering gender interaction term as well as a volunteering education interaction term was included. The added interaction terms did not identify any significant effects. Additional file 4 depicts detailed results of the sensitivity analysis with specialist visits as outcome measure since solely for specialist visits a significant effect has been found in the main model.

## Discussion

### Main findings

In the present study, we used data from a representative sample of adults older than 40 years in Germany to investigate whether volunteering is associated with health care utilization, covering GP and specialist visits as well as nights in the hospital longitudinally. Pairwise correlations revealed that volunteering is correlated with GP visits. After controlling for possible confounders, the results of our retrospective analyses indicate that the number of specialist visits increased with changes in volunteering, whereas GP visits and hospitalization were not affected by volunteering. Besides, all three outcome measures were significantly associated with self-rated health. For sensitivity analysis, we tested whether gender or educational level moderate the effect of volunteering on health care utilization. None of the interaction terms did achieve statistical significance.

### Relation to previous research

Since the majority of the existing literature is based on physical and mental health-related outcomes rather than patterns of health care utilization, it is difficult to compare our results with preceding studies. To the best of our knowledge, Kim and Konrath [22] were the first researchers who analyzed the association between volunteering and health care utilization. After controlling for a number of covariates, they found that volunteers have a greater probability to use preventive health care services and spend a lower number of nights in the hospital compared to those who do not volunteer. They did not find a significant association between volunteering and the frequency of doctor visits. Compared to our study, findings were partly different. This might be due to differences in the design and structure of the studies. By analyzing intra-individual changes over time, our study extends current knowledge [22]. The existing study focused on models that compare individuals who volunteer with non-volunteers. This might lead to an overestimation of the impact of volunteering on health care utilization since they did not control for (time-constant) unobserved heterogeneity. Thus, drawing conclusions about causal effects might be difficult. Moreover, they did not distinguish between different medical specialties, even if the predictors are very likely to be influenced by that.

There are a number of studies looking at more general concepts of volunteering, such as social relationships or social interactions that aimed at analyzing an association with health care use [44, 45]. It might be that these concepts are somewhat related to volunteering. As they are not completely congruent with formal volunteering itself, we refrained from comparing them with our findings.

Initially, we hypothesized that volunteering is negatively associated with health care utilization because of the positive health related outcomes linked with the volunteer involvement of older adults. We could not confirm this hypothesis. There are several possible explanations for more visits to specialists resulting from changes in the involvement in volunteering. For example, people who volunteer are characterized by having more empathic traits than non-volunteers [46, 47]. Consequently, volunteers might be more concerned about how their poor health status affects the well-being of others and therefore, they are interested in staying healthy. This in turn could lead to an increase in the use of health treatments, such as preventive services. This explanation is in line with Kim and Konrath [22]. However, this explanation is based on the assumption that preventive services are often performed by specialists, e.g., cervix screening at gynecologists [48]. Another possible explanation is that volunteering appears to ease access to health-related information [13, 49]. This might alert people to behave more health-conscious and could in turn lead to a higher amount of specialist visits, again due to increases in preventive services. Thereby, it is likely that people gather health-related information particularly through voluntary activities which are related to areas of health (e.g., volunteering in hospitals). Additionally, volunteers are not only concerned about others but are likely to have a strong sense of their own value. For example, Okun [50] found that volunteering is related with a higher level of self-esteem. People who volunteer might care about their health by using more preventive services. This might be a possible explanation of the higher rate of specialist consultations of the volunteers. Moreover, being involved in volunteering can lead to psychological stress and burnout. Scientists found that volunteering within onerous areas, such as volunteering in hospices, can cause psychological stress which may lead to the abandonment of the voluntary work [51]. Thus, the increase of specialist visits could result from more consultations with, e.g., neurologists. According to previous research, volunteering was found to be positively associated with physical activities [13]. The higher amount of specialist visits might stem from injuries or accidents resulting from those physical activities (e.g., orthopedists). Moreover, it was shown that the number of contacts with specialists increases significantly with higher educational level [52]. As we already mentioned, there is a positive association between the level of education and the probability to volunteer [39], which supports our findings.

However, as there is evidence that volunteering is associated with better physical and mental health, it is somewhat surprising that no statistically significant results were found for GP visits nor hospitalization in the FE regression analyses. It is difficult to explain why solely associations with the number of specialist visits were significant but not with GP visits considering that people generally contact GPs first as they often operate as gatekeeper and coordinators [44]. Free accessibility to physicians of all medical specialties, as it is usually the case in Germany, might explain these findings. Additionally, scientists found that people suffering from multi-morbidities contact more medical specialties [53]. As our data set consists of older adults who are generally more likely to have complex diseases and multi-morbidities [54], this could confirm our explanation. It might be that these diseases are sufficiently treated by specialists and therefore, hospitalization is not required. However, it is worth noticing that these explanations are only of theoretical nature and based on the assumption that serious illnesses generally require the involvement of (several) specialists.

### Strengths and limitations

This is the first study examining the impact of volunteering on the use of health care services in Germany. Data were derived from a large, population-based study of community-dwelling older individuals. By using panel data methods (FE regressions), we provide a means of controlling the impact of time-constant unobserved factors. Another strength refers to the distinction between GPs and specialists as literature found educational and health-related differences in the use of GPs and specialists [52].

It is worth noting that the FE estimator uses only within-variation of the individuals. Thus, our findings refer to individuals with changes in the use of health care services during the given time period (average treatment effect of the treated) [35]. We cannot dismiss the possibility that estimates might be biased due to reverse causation. For example, it could be possible that after being treated by a specialist, e.g., for reasons of a complex disease, individuals seek to give something back and to be meaningfully active. For this purpose, volunteering could be a good venue. The application of panel instrumental variables methods to address the endogeneity problem, require strong assumptions [34]. Therefore, FE regressions were used in this study. It is important to take potential sample selection bias and panel attrition into account. However, both effects were shown to be small in the German Ageing Survey [27] and it has been demonstrated that panel attrition is not necessarily an issue when selected longitudinal aging studies examine the association between variables [55, 56]. Moreover, we cannot dismiss the possibility that other time-varying factors (e.g., changes in accessibility) exist that bias our estimates. Future research is required to clarify this issue. Lastly, for reasons of data unavailability, our analysis was restricted to three waves with relatively long time spans between waves. As a result*,* short-term changes might not be recognized. Also, due to data unavailability, it was not possible to include further lifestyle factors, such as alcohol consumption or physical activities. We could neither distinguish between the specific type or intensity of the voluntary activity nor between different forms of health insurance (statutory and private health insurance) due to reasons of data availability. Future research is required to clarify these issues.

## Conclusion and future research

The number of older people is predicted to increase in the upcoming decades and societal aging is assumed to be a challenge for public economies as well as health care systems. Notwithstanding, the growing number of active and healthy older adults may provide new opportunities, e.g., through volunteering. Volunteering is recognized as a promising approach within the larger context of societal aging, as it may not only contribute positively to society but also to the individuals themselves. In sum, these facts underline the importance of analyzing the impact of volunteering. By investigating the effects of volunteering on the use of health care services in older age, we aimed to advance knowledge about its impact. In our study, volunteering was identified to be associated with increases in the use of specialist visits, but not with hospitalization and the amount of GP visits of older adults in Germany.

Continually investigating the association between volunteering and health care utilization will enhance the knowledge and understanding within this research field. This might be reasonable in promoting comprehensive ways for volunteering and developing social programs and policies. As already mentioned, to date, research is sparse and more detailed information regarding the specific context of the voluntary activities is necessary to understand the link and the underlying mechanisms between volunteering and health care utilization. It is most likely that the impact of volunteering on the use of health care services is not only linked to the role but also to the intensity to which people are involved. Results of the study by Musick and Wilson [15] revealed that church-related volunteering affected depression to a greater extent than secular volunteering. It was also shown that the beneficial effects of volunteering on health are non-linear and that the time commitment of the volunteer is decisive. In that matter, Luoh and Herzog [13] found the beneficial effects of volunteering to decrease with a volunteering level of more than 100 h per year. It might also be crucial for future studies to include personality traits since researchers found that people with certain personal characteristics are more likely to volunteer [57]. In line with this, volunteering is additionally assumed to have different meanings for people with various cultural or ethnical backgrounds [58]. The consideration of such differences might be reasonable in order to account for the growing diversity of the older population. Future studies could include those time-constant factors, for example, in terms of moderator variables in regression analysis.

## Additional files

Additional file 1: Composition of specialists. (DOCX 13 kb)

Additional file 2: Hausman’s specification test. (DOCX 13 kb)

Additional file 3: Pairwise correlation Matrix (with Bonferroni correction for multiple comparisons). (XLSX 41 kb)

Additional file 4: Results of FE poisson regressions (interaction terms with specialist visits as outcome measure; waves 2–4). (DOCX 16 kb)

## Abbreviations

BMFSFJ: German Federal Ministry for Family Affairs, Senior Citizens, Women, and Youth
BMI: Body Mass Index
CAPI: Computer-Assisted Personal Interview
DEAS: German Ageing Survey
DZA: German Centre of Gerontology
FE: Fixed Effects
FWS: German Survey on Volunteering
GP: General Practitioner
ISCED: International Standard Classification of Education
OECD: Organization for Economic Co-operation and Development
OR: Odds Ratio
PHI: Private Health Insurance
POLS: Pooled Ordinary Least Square
RE: Random Effects
SD: Standard Deviation
SHI: Statutory Health Insurance
WHO: World Health Organization

## References

[1] Simonson J, Vogel C, Tesch-Römer C. Freiwilliges Engagement in Deutschland*: Der Deutsche Freiwilligensurvey*. 2014:2014.

[2] European Year of Volunteering (2011). *Policy* Agenda *for Volunteering in Europe (PAVE)*. EYV 2011 Alliance.

[3] United Nations Volunteers (Hrsg.) (2011). *State of the world*’*s volunteerism report* 2011*. Universal values for global well-being*. United Nations Volunteers.

[4] Wilson J, Musick M (1997). Who cares? Toward an integrated theory of volunteer work. Am Sociol Rev.

[5] Morrow-Howell N, Hong SI, Tang F (2009). Who benefits from volunteering? Variations in perceived benefits. The Gerontologist.

[6] Spijker J, MacInnes J (2013). Population ageing: the timebomb that isn’t?. BMJ.

[7] Choi NG, Burr JA, Mutchler JE, Caro FG (2007). Formal and informal volunteer activity and spousal caregiving among older adults. Research on aging.

[8] Morrow-Howell N (2010). Volunteering in later life: research frontiers. J Gerontol: Series B.

[9] Casiday R, Kinsman E, Fisher C, Bambra C (2008). Volunteering and health: what impact does it really have. London: Volunteering England.

[10] Greenfield EA, Marks NF (2004). Formal volunteering as a protective factor for older adults’ psychological well-being. J Gerontol Ser B Psychol Sci Soc Sci.

[11] Wu AM, Tang CS, Yan EC (2005). Post-retirement voluntary work and psychological functioning among older Chinese in Hong Kong. J Cross-Cultural Gerontol.

[12] Windsor TD, Anstey KJ, Rodgers B (2008). Volunteering and psychological well-being among young-old adults: how much is too much?. The Gerontologist.

[13] Luoh MC, Herzog AR (2002). Individual consequences of volunteer and paid work in old age: health and mortality. J Health Soc Behav.

[14] Lum TY, Lightfoot E (2005). The effects of volunteering on the physical and mental health of older people. Res Aging.

[15] Musick MA, Wilson J (2003). Volunteering and depression: the role of psychological and social resources in different age groups. Soc Sci Med.

[16] Li Y, Ferraro KF (2005). Volunteering and depression in later life: social benefit or selection processes?. J Health Soc Behav.

[17] Van Willigen M (2000). Differential benefits of volunteering across the life course. J Gerontol Ser B Psychol Sci Soc Sci.

[18] Baker LA, Cahalin LP, Gerst K, Burr JA (2005). Productive activities and subjective well-being among older adults: the influence of number of activities and time commitment. Soc Indic Res.

[19] Musick MA, Herzog AR, House JS (1999). Volunteering and mortality among older adults: findings from a national sample. J Gerontol B Psychol Sci Soc Sci.

[20] Shmotkin D, Blumstein T, Modan B (2003). Beyond keeping active: concomitants of being a volunteer in old-old age. Psychol Aging.

[21] Harris AH, Thoresen CE (2005). Volunteering is associated with delayed mortality in older people: analysis of the longitudinal study of aging. J Health Psychol.

[22] Kim ES, Konrath SH (2016). Volunteering is prospectively associated with health care use among older adults. Soc Sci Med.

[23] Passon A, Lüngen M, Gerber A, Redaelli M, Stock S. Das Krankenversicherungssystem in Deutschland. Gesundheitsökonomie. 2009:105–36.

[24] Van Doorslaer E, Masseria C, Koolman X (2006). Inequalities in access to medical care by income in developed countries. Can Med Assoc J.

[25] Mahne K, Wolff JK, Simonson J, Tesch-Römer C (2017). Altern im Wandel: Zwei Jahrzehnte Deutscher Alterssurvey.

[26] Schiel S, Dickmann C, Aust F (2011). Methodenbericht Deutscher Alterssurvey (DEAS): 4. Befragungswelle.

[27] Klaus D, Engstler H, Mahne K, Wolff JK, Simonson J, Wurm S, Tesch-Römer C (2017). Cohort profile: the German ageing survey (DEAS). Int J Epidemiol.

[28] Bock JO, Hajek A, Koenig HH (2017). The longitudinal association between psychological factors and health care use. Health Serv Res.

[29] Andersen RM (1995). Revisiting the behavioral model and access to medical care: does it matter?. J Health Soc Behav.

[30] Babitsch, B., Gohl, D., & von Lengerke, T. (2012). Re-revisiting Andersen’s behavioral model of health services use: a systematic review of studies from 1998-2011*.* Psychosoc Med*,* 9, Doc11: 1–15. 10.3205/psm000089 PMC3488807 23133505

[31] Unesco, ed. (2006). International Standard Classification of Education ISCED 1997. Re-edition. Paris: UNESCO.

[32] Charlson M, Szatrowski TP, Peterson J, Gold J (1994). Validation of a combined comorbidity index. J Clin Epidemiol.

[33] WHO (2006). BMI classification. World Health Organization (WHO). Geneva, Switzerland. [Online]. Available: http://apps.who.int/bmi/index.jsp?introPage=intro_3.html. [Accessed 11.03.2018].

[34] Cameron AC, Trivedi PK (2005). Microeconometrics: methods and applications.

[35] Brüderl J, Ludwig V, Best H (2015). Fixed-effects panel regression. The SAGE handbook of regression analysis and causal inference.

[36] Hausman JA (1978). Specification tests in econometrics. Econometrica: Journal of the econometric society.

[37] Stock JH, Watson MW (2008). Heteroscedasticity-robust standard errors for fixed effects panel data regression. Econometrica.

[38] Allison PD (2009). Fixed effects regression models.

[39] Wilson J (2000). Volunteering. Annu Rev Sociol.

[40] Helms S, McKenzie T (2014). Gender differences in formal and informal volunteering in Germany. Volunt Int J Volunt Nonprofit Org.

[41] Corp S. Stata statistical software: release 14. Texas: Stata Corp LP: College Station. 2015.

[42] Cameron AC, Trivedi PK (2010). Microeconometrics using Stata (rev. ed.). College Station.

[43] Mukaka MM (2012). A guide to appropriate use of correlation coefficient in medical research. Malawi Med J.

[44] Bremer, D., Lüdecke, D., Vonneilich, N., & von dem Knesebeck, O. (2018). Social relationships and GP use of middle-aged and older adults in Europe: a moderator analysis. BMJ Open*,* 8*(*4*)*, 1–12. 10.1136/bmjopen-2017-018854 PMC5892736 29627805

[45] Valtorta NK, Moore DC, Barron L, Stow D, Hanratty B (2018). Older adults’ social relationships and health care utilization: *A systematic review*. Am J Public Health.

[46] Musick MA, Wilson J (2008). Volunteers: a social profile.

[47] Wilhelm MO, Bekkers R (2010). Helping behavior, dispositional empathic concern, and the principle of care. Soc Psychol Q.

[48] Lorant V, Boland B, Humblet P, Deliège D (2002). Equity in prevention and health care. J Epidemiol Community Health.

[49] House JS, Landis KR, Umberson D (1988). Social relationships and health. Science.

[50] Okun MA (1994). The relation between motives for organizational volunteering and frequency of volunteering by elders. J Appl Gerontol.

[51] Claxton-Oldfield S, Claxton-Oldfield J (2012). Should I stay or should I go: a study of hospice palliative care volunteer satisfaction and retention. Am J Hosp Palliat Care®.

[52] Geling O, Janssen C, Lüschen G (1996). Alter, Gesundheitsstatus und die Inanspruchnahme von Allgemein-und Fachärzten. Sozial-und Präventivmedizin.

[53] Thode N, Bergmann E, Kamtsiuris P, Kurth BM (2005). Einflussfaktoren auf die ambulante Inanspruchnahme in Deutschland. Bundesgesundheitsblatt Gesundheitsforsch Gesundheitsschutz.

[54] Kohler M, Ziese T. Telefonischer Gesundheitssurvey des Robert Koch-Instituts zu chronischen Krankheiten und ihren Bedingungen: deskriptiver Ergebnisbericht: Beiträge zur Gesundheitsberichterstattung des Bundes: Robert Koch-Institut; 2004.

[55] Kempen GI, van Sonderen E (2002). Psychological attributes and changes in disability among low-functioning older persons: does attrition affect the outcomes?. J Clin Epidemiol.

[56] Wurm S, Tesch-Römer C, Tomasik MJ (2007). Longitudinal findings on aging-related cognitions, control beliefs, and health in later life. J Gerontol B Psychol Sci Soc Sci.

[57] Carlo G, Okun MA, Knight GP, de Guzman MRT (2005). The interplay of traits and motives on volunteering: agreeableness, extraversion and prosocial value motivation. Personal Individ Differ.

[58] Johnson KJ, Lee SH (2017). Factors associated with volunteering among racial/ethnic groups: findings from the California health interview survey. Res Aging.

