# Supplementary material for: The association between voluntary work and health care use among older adults in Germany
Source: BMC Health Serv Res. 2019 Jan 15;19:39. doi: 10.1186/s12913-019-3867-x (PMC6334381; doi:10.1186/s12913-019-3867-x)
Supplement: Supplementary file 1 — Composition of specialists. (DOCX 13 kb) [file 12913_2019_3867_MOESM1_ESM.docx]

| **Specialists** | **Medical specialty** |
| --- | --- |
|  | Internists |
|  | Gynecologists |
|  | Dentists |
|  | Ophthalmologists |
|  | Orthopedists |
|  | Ear, nose, and throat specialists |
|  | Neurologists |
|  | Psychiatrists |
|  | Dermatologists |
|  | Urologists |
|  | Other specialists (open answer) |

Additional file 1: Composition of specialists
