# Supplementary material for: The association between voluntary work and health care use among older adults in Germany
Source: BMC Health Serv Res. 2019 Jan 15;19:39. doi: 10.1186/s12913-019-3867-x (PMC6334381; doi:10.1186/s12913-019-3867-x)
Supplement: Supplementary file 2 — Hausman’s specification test. (DOCX 13 kb) [file 12913_2019_3867_MOESM2_ESM.docx]

| Outcome measure | Hausman test statistic | p-value |
| --- | --- | --- |
| Hospitalization | 30.56 | P < 0.01 |
| GP visits | 143.5 | P < 0.001 |
| Specialist visits | 304.85 | P < 0.001 |

Additional file 2: Hausman's specification test
