# Supplementary material for: The association between voluntary work and health care use among older adults in Germany
Source: BMC Health Serv Res. 2019 Jan 15;19:39. doi: 10.1186/s12913-019-3867-x (PMC6334381; doi:10.1186/s12913-019-3867-x)
Supplement: Supplementary file 4 — Results of FE poisson regressions (interaction terms with specialist visits as outcome measure; waves 2–4). (DOCX 16 kb) [file 12913_2019_3867_MOESM4_ESM.docx]

Additional file 4: Results of FE poisson regressions (interaction terms with specialist visits as outcome measure; waves 2-4)

|  |  | **Specialist visits Interaction with gender** | **Specialist visits Interaction with education** |  |
| --- | --- | --- | --- | --- |
| **Predisposing factors** | Other marital statuses (ref.: Married, living together with spouse) | -0.0049 (0.0578) | -0.0262 (0.0592) |  |
|  | Retired (ref.: Working) | -0.0117 (0.0455) | 0.0065 (0.0466) |  |
|  | Other: not employed | 0.0845+ (0.0449) | 0.0972* (0.0457) |  |
|  | Age (in years) | -0.0086** (0.0029) | -0.0083** (0.0030) |  |
| **Enabling resources** | (Log) monthly equivalent net income | 0.0471 (0.0378) | 0.0525 (0.0379) |  |
|  | Self-rated accessibility of doctors and pharmacies (ref.: No accessibility) | 0.0052 (0.0291) | -0.0002 (0.0295) |  |
| **Need factors** | Underweight (ref.: Normal weight) | -0.0568 (0.1598) | -0.0558 (0.1595) |  |
|  | Overweight | -0.0650+ (0.0385) | -0.0884* (0.0385) |  |
|  | Obesity | -0.0445 (0.0621) | -0.0623 (0.0628) |  |
|  | Self-rated health (from “very good” to “very bad”) | 0.1449*** (0.0175) | 0.1471*** (0.0176) |  |
|  | Number of chronic diseases | 0.0596*** (0.0091) | 0.0581*** (0.0093) |  |
|  | Currently smoking (ref.: Currently not smoking) | -0.0777 (0.0570) | -0.0741 (0.0572) |  |
| **Volunteering** | Volunteer involvement (ref.: No volunteer involvement) | 0.0547 (0.0423) | 0.3164+ (0.1622) |  |
|  | Voluntary involvement##gender (ref.: Male) | 0.016 (0.0597) |  |  |
|  | Volunteer involvement##medium education (3–4) (ref.: Low education (1-2)) |  | -0.2375 (0.1683) |  |
|  | Volunteer involvement##high education  (5-6) (ref.: Low education (1-2)) |  | -0.2943+ (0.1671) |  |
|  | Observations | 7,068 | 6,816 |  |
|  | Number of individuals | 3,233 | 3,107 |  |
| Notes: Beta coefficients were reported; Cluster-robust standard errors in parentheses;  Ref. = Reference;  *** p < 0.001, ** p < 0.01, * p < 0.05, + p < 0.10 | | | | |
|  |  |  |  |  |
|  |  |  |  |  |
|  |  |  |  |  |
